# Correlation between Inflammatory Systemic Biomarkers and Surgical Trauma in Elderly Patients with Hip Fractures

**DOI:** 10.3390/jcm12155147

**Published:** 2023-08-06

**Authors:** Flaviu Moldovan, Adrian Dumitru Ivanescu, Pal Fodor, Liviu Moldovan, Tiberiu Bataga

**Affiliations:** 1 Orthopedics—Traumatology Department, Faculty of Medicine, “George Emil Palade” University of Medicine, Pharmacy, Science, and Technology of Targu Mures, 540142 Targu Mures, Romania; adrian.ivanescu@umfst.ro (A.D.I.); pal.fodor@umfst.ro (P.F.); tiberiu.bataga@umfst.ro (T.B.); 2 Department of Training, Technological Innovation, and Research in Orthopedics-Traumatology, “George Emil Palade” University of Medicine, Pharmacy, Science, and Technology of Targu Mures, 540142 Targu Mures, Romania; 3 Faculty of Engineering and Information Technology, “George Emil Palade” University of Medicine, Pharmacy, Science, and Technology of Targu Mures, 540142 Targu Mures, Romania; liviu.moldovan@umfst.ro

**Keywords:** hip fractures, extracapsular, intracapsular, gamma nail, bipolar hemiarthroplasty, neutrophil-to-lymphocyte ratio, platelet-to-lymphocyte ratio, monocyte-to-lymphocyte ratio, systemic immune-inflammation index

## Abstract

The treatment for hip fractures consists of a wide variety of orthopedic implants ranging from prosthesis to intramedullary nails. The purpose of this study is to determine the correlation between blood-count-derived biomarkers such as the neutrophil-to-lymphocyte ratio (NLR), the platelet-to-lymphocyte ratio (PLR), the monocyte-to-lymphocyte ratio (MLR) and the systemic immune-inflammation index (SII) and the level of aggression sustained by elderly patients during these surgical procedures. A total of 129 patients aged over 70 and diagnosed with acute hip fractures who underwent surgical treatment between November 2021 and February 2023 were included in our observational retrospective cohort study. Two groups were formed depending on the anatomic location of the fracture for statistical comparison: group 1 with extracapsular fractures, who received a closed reduction internal fixation (CRIF) with a gamma nail (GN) as treatment, and group 2 with intracapsular fractures, who received a bipolar hemiarthroplasty (BHA) as treatment. The length of hospital stay (LHS), duration of surgery, preoperative days, pre- and postoperative red blood count (RBC) and hemoglobin (HGB) levels and postoperative NLR, PLR and SII were significantly different between the two groups (*p* < 0.05). Furthermore, the multivariate analysis indicated that the postoperative NLR (*p* = 0.029), PLR (*p* = 0.009), SII (*p* = 0.001) and duration of surgery (*p* < 0.0001) were independently related to the invasiveness of the procedures. The ROC curve analysis demonstrated that a postoperative SII > 1564.74 is a more reliable predictor of surgical trauma in terms of specificity (58.1%) and sensitivity (56.7%). Postoperative SII as a biomarker appears to be closely correlated with surgical trauma sustained by an older population with hip fractures.

## 1. Introduction

Hip fractures are one of the most frequent injuries in the elderly population, and they are associated with high health care costs and substantial morbidity and mortality [1]. It is estimated that the incidence of these fractures will increase from 1.66 million in 1990 to 6.26 million by 2050 [2]. In terms of surgical treatment, the anatomic location pays an important role in dictating the type of procedure needed. Intracapsular fractures consist of subcapital and transcervical subgroups, with hemiarthroplasty being one of the best choices of treatment for frail patients. Extracapsular fractures occur distal to the attachment of the joint capsule to the femur and can be divided into basicervical, intertrochanteric and pertrochanteric subgroups, with osteosynthesis being achieved with an intramedullary nail through a closed reduction technique under intraoperative fluroscopic control [3].

Different measurements such as operative time, preoperative stay, length of hospitalization, blood loss, length of incision and postoperative pain scores are used to compare the invasiveness of various procedures in multiple medical fields [4,5,6]. However, a defensive inflammatory response can be triggered due to injury, infection or ischemia [7], which can lead to increased platelets (PLTs) as they are the central actors of this response [8]. An increased leukocyte count was also linked to having a predictive role in stress and trauma [9]. It is well known that neutrophils contribute to bone fracture healing, as their levels rise in the early fracture hematoma, initiating the downstream process of the primary immune response. They also have an important role in the removal of damage-associated molecular patterns (DAMPs) and microbe- or pathogen-associated molecular patterns (MAMPs) in unsterile skin wounds and can enhance the healing process [10]. Osteoclasts are differentiated in the course of callus formation from monocytes that are engaged to the fracture site [11]. High-energy injuries like road traffic accidents and falls from height are associated with lymphopenia, which contributes to increased mortality rates and longer hospital stays as Guo et al. demonstrated [12].

Complete blood counts (CBCs) are routinely performed for all patients at admission and on the first day after any surgical procedure. As a consequence, simple and economic predictive hematologic biomarkers like the neutrophil-to-lymphocyte ratio (NLR), the platelet-to-lymphocyte ratio (PLR), the monocyte-to-lymphocyte ratio (MLR) and the systemic immune-inflammation index (SII) can easily be obtained [13,14].

Recently, these biomarkers have been correlated with systemic inflammation sustained by the human organism after several types of fractures, polytraumatisms, carcinomas and various musculoskeletal disorders. Their diagnostic and prognostic values have been linked to various surgery-related fields. In the early stages of colorectal cancer [15], these biomarkers can also be successfully used for screening. MLR as a specific marker was correlated with patients diagnosed with non-Hodgkin and Hodgkin lymphomas [16]. Physicians are now more aware of the immune responses that are triggered postoperatively and their potential negative outcomes. Tzikos et al. [17] demonstrated the efficiency of NLR and PLR indexes as predicting factors for 90-day mortality and the length of hospital stay in patients undergoing cardiac surgery. Another study by Parmana et al. [18] showed that elevated preoperative SII values after off-pump coronary artery bypass graft surgery can anticipate intensive care unit stay and prolonged mechanical ventilation. In the field of orthopedic trauma, there are only a few studies on this topic. One example is the research conducted by Wang et al. [19]. They compared the invasiveness of two osteosynthesis procedures in terms of inflammation for high-energy bicondylar tibial plateau fractures (Schatzker types V and VI) in younger patients using postoperative NLR and PLR indexes.

As far as we know, there are no data for the use of these hematologic indexes in predicting the surgical trauma of different operative protocols sustained by patients with hip fractures [20]. Hence, the objective of this study is to perform a retrospective cohort study to identify any associations between NLR, PLR, MLR and SII ratios and the invasiveness of the two interventions proposed.

## 2. Materials and Methods

### 2.1. Study Design and Patients

**Figure 1:**
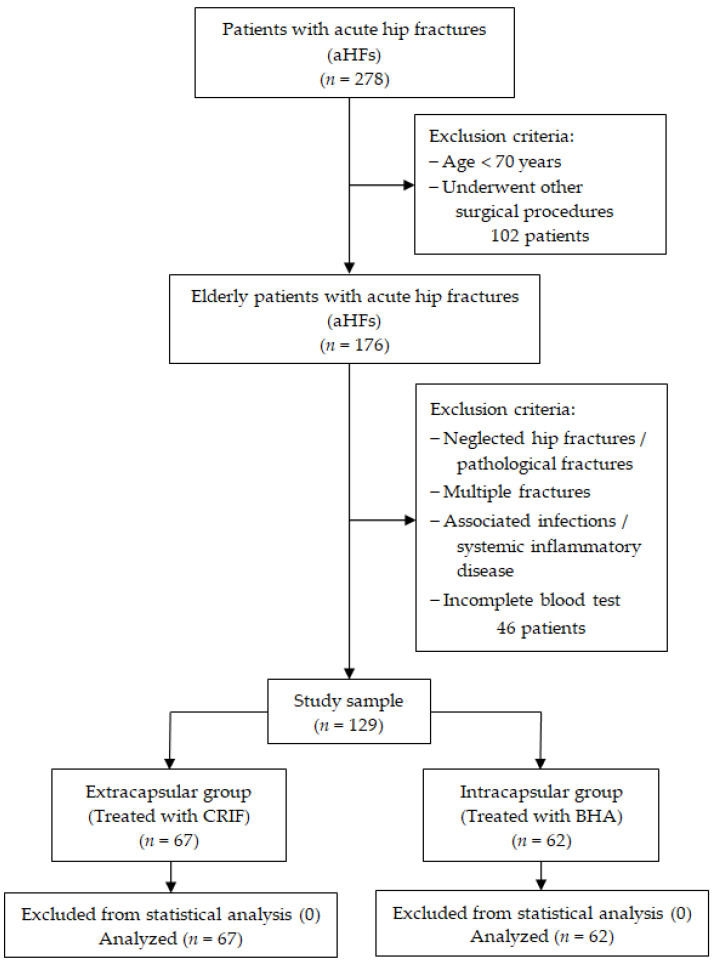
Flow diagram for selection of the study population.

An observational retrospective cohort study was performed at the Orthopedics-Traumatology Department of Mures County Emergency Hospital in Targu Mures, Romania between November 2021 and February 2023. The study was conducted in accordance with the Declaration of Helsinki and was approved under the protocol code Ad.22522/17 in September 2021 by the Ethics Committee of the hospital. We included patients diagnosed with acute hip fractures and aged over 70 who underwent either a bipolar hemiarthroplasty (BHA) or a CRIF with gamma nail (GN) as treatment, depending on the location of the fracture. Exclusion criteria from the study were patients younger than 70 years of age, patients with neglected hip fractures or with pathologic fractures, those with multiple fractures, patients with associated infectious or systemic inflammatory diseases and patients with incomplete blood tests. We initially formed two groups of patients: those with intracapsular fractures (treated with BHA, *n* = 62) and those with extracapsular fractures (treated with GN, *n* = 67). The flowchart of the patient screening process is presented in Figure 1.

### 2.2. Data Collection

From the hospital’s computerized database, we collected the following data [21,22]: (1) age, sex and living area; (2) lifestyle risk factors such as tobacco use, alcohol use and obesity (BMI > 25); (3) medical history (high blood pressure—HBP, asthma or chronic obstructive pulmonary disease—COPD, chronic venous insufficiency—CVI, congestive heart failure—CHF and diabetes mellitus—DM); (4) surgery-related data such as preoperative days, duration of surgery (minutes), type of anesthesia, American Society of Anesthesiologists (ASA) score and length of hospital stay (LHS); (5) laboratory tests at admission and at 1st postoperative day including neutrophil count, lymphocyte count, monocyte count, platelet (PLT) count, aspartate transaminase/alanine transaminase (AST/ALT) ratio, white blood count (WBC), red blood count (RBC) and hemoglobin (HGB) level.

### 2.3. Inflammatory Systemic Biomarkers

In the next stage of the research, we computed the four inflammatory biomarkers in order to test their correlation with surgical trauma of the two proposed hip fracture interventions. 

The first marker, the neutrophil-to-lymphocyte ratio (NLR), is defined as the report between the neutrophil count and lymphocyte count, expressed by the subsequent formula

NLR = neutrophil count/lymphocyte count

The second marker, the platelet-to-lymphocyte ratio (PLR), is defined as the report between the platelet count and lymphocyte count, in the following formula:

PLR = platelet count/lymphocyte count

The next marker, the monocyte-to-lymphocyte ratio (MLR), is defined as the report between the monocyte count and lymphocyte count, as follows:

MLR = monocytes count/lymphocyte count

And finally, the fourth marker, the systemic immune-inflammation index (SII), is defined as the product between the neutrophil count and platelet count divided by the lymphocyte count, as stated in the following formula:

SII = (neutrophile count × platelet count)/lymphocyte count

### 2.4. Surgical Technique and Postoperative Care

All procedures were performed using the same technique by experienced orthopedic surgeons from the research team. Patients underwent general or regional intraspinal anesthesia and were given standard antibiotic prophylaxis with Cefuroxime 1.5 g for 3 days. For the femoral neck fractures (intracapsular group), cemented bipolar hemiarthroplasty (BHA) was performed through an antero-lateral Watson-Jones approach, with the length of incision being approximately 12–14 cm. As for the intertrochanteric fractures (extracapsular group), a closed reduction on a dedicated orthopedic table (that ensured the adequate traction and correction of the vicious position of the leg) and internal fixation under intraoperative fluoroscopy using a gamma nail (GN) was performed, with the length of incision being approximately 3–4 cm depending on the BMI of the patient.

### 2.5. Statistical Analysis

For the statistical analysis, we used SPSS (version 28.0.1.1) for Windows (SPSS, Inc., Chicago, IL, USA).

Continuous variables were analyzed using Student’s *t* test or Mann–Whitney U test, depending on their normality check. Categorical variables were assessed for intergroup significant differences using Chi-squared test or Fisher’s exact test. For the biomarkers and the admissible variables, the receiver operating characteristic (ROC) curve analysis was used to determine the cut-off values based on the Youden index (Youden index = sensitivity + specificity − 1, ranging from 0 to 1) and the predictive power. In addition, to identify independent predictors of surgical trauma, a multiple logistic regression analysis was performed, taking into account the postoperative hematologic indexes, associated risk factors and relevant indexes. This model was indicated as being acceptable using the Hosmer–Lemeshow test with a *p* value > 0.05.

## 3. Results

A total of 129 elderly patients (72.9% female) aged over 70 years that sustained acute hip fractures were included in a 16-month study period. They were further divided into the following two groups depending on the anatomic location of the fracture: the extracapsular group with 67 patients (51.9%), who underwent a CRIF with gamma nail (GN) as treatment, and the intracapsular group with 62 patients (48.1%), who underwent a bipolar hemiarthroplasty (BHA) as treatment.

First, the cut-off points (Table 1) of the relevant variables, including the preoperative days, length of hospital stay, duration of surgery and the inflammatory systemic biomarkers (NLR, PLR, MLR, and SII) at admission and post operation were determined with the use of an ROC curve analysis. 

**Figure 2:**
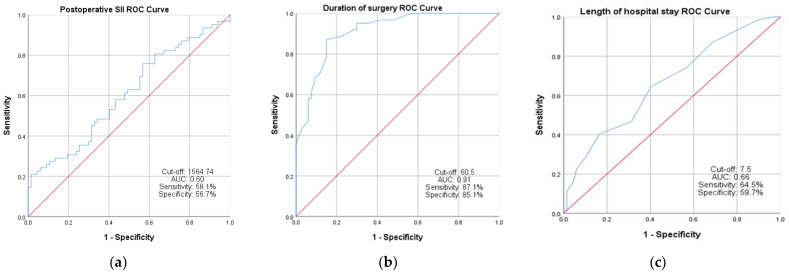
ROC curve indicating the ideal cut-off values, and the AUC (area under the curve), sensitivity and specificity of (**a**) postoperative SII, (**b**) duration of surgery in minutes and (**c**) length of hospital stay for intracapsular versus extracapsular hip fractures.

The predictive ability was determined by calculating the AUC, sensitivity and specificity (Figure 2) for the postoperative SII (cut-off, 1564.74; AUC, 0.60; sensitivity, 58.1%; specificity, 56.7%), duration of surgery (cut-off, 60.5; AUC, 0.91; sensitivity, 87.1%; specificity, 85.1%) and LHS (cut-off, 7.5; AUC, 0.66; sensitivity, 64.5%; specificity, 59.7%).

The differences between the extracapsular group and intracapsular group (Table 2) were in the alcohol use (*p* = 0.045), the baseline characteristics of the patients and the preoperative days (*p* = 0.022), the LHS (*p* = 0.008) and the duration of surgery (*p* < 0.0001) for the surgery-related data. Regarding the laboratory data, we identified significant differences at admission for the monocyte count (*p* = 0.038), RBC (*p* < 0.0001), HBG level (*p* < 0.0001) and postoperative neutrophil count (*p* = 0.047), RBC (*p* < 0.0001), HGB level (*p* < 0.0001), NLR (*p* = 0.041), PLR (*p* = 0.014) and SII (*p* = 0.012). 

**Figure 3:**
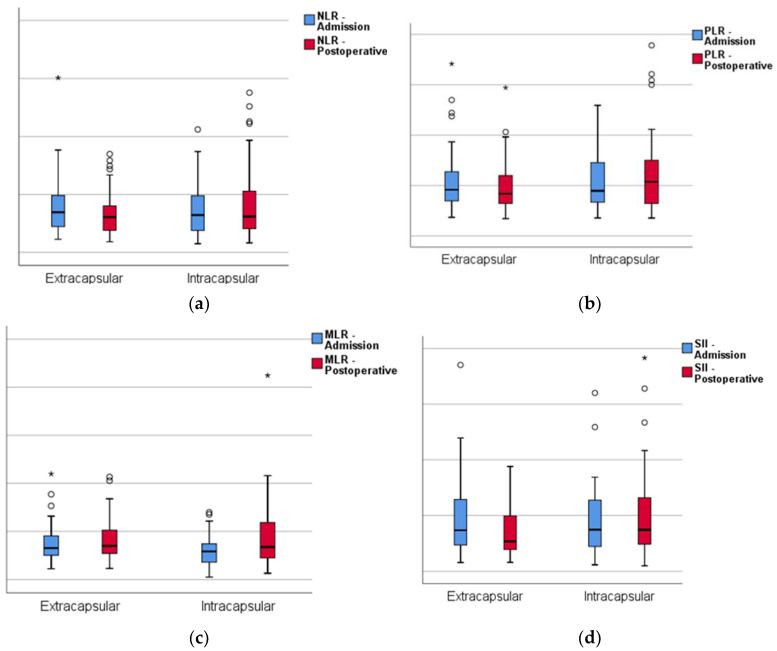
Boxplots of the two groups at admission and after surgery: (**a**) neutrophil-to-lymphocyte ratio; (**b**) platelet-to-lymphocyte ratio; (**c**) monocyte-to-lymphocyte ratio; (**d**) systemic immune-inflammation index. Circles represent the mild outliers and asterisks represent the extreme outliers.

Furthermore, the inflammatory markers (NLR, PLR, MLR and SII) were significantly higher post operation compared to the admission period (Figure 3).

In the multivariate logistic regression analysis, we included the duration of the surgery, the type of anesthesia used and the associated risk factors such as alcohol use, tobacco use, obesity, HBP, asthma and COPD, CVI, CHF, CHD and DM that may influence the immediate systemic immune response of the patients after surgery.

The outcomes **(**Table 3) that suggested an association with the invasiveness of the procedure performed were the NLR (OR 1.79, 95% CI 0.56–2.30, *p* = 0.029), PLR (OR 2.26, 95% CI 1.07–4.77, *p* = 0.009), SII (OR 2.49, 95% CI 1.22–5.07, *p* = 0.001) and the duration of surgery (OR 38.47, 95% CI 14.13–104.73, *p* < 0.0001).

The Hosmer and Lemeshow test showed a good fitness (X^2^ = 4.464, *p* = 0.812, Nagelkerke R^2^ (*p* = 0.744)).

## 4. Discussion

According to recent studies, inflammatory systemic biomarkers have been used in the field of orthopedics to assess the severity of different types of fractures [23,24] and to predict complications after surgeries such as periprosthetic joint infections [25] and acute deep vein thrombosis [26]. In the present study, we compared the magnitude of two commonly performed orthopedic interventions in order to identify any associations with these inexpensive, easy-to-obtain markers. The outcomes of the multivariate logistic regression showed that the postoperative NLR (OR 1.79, 95% CI 0.88–3.64, *p* = 0.029), PLR (OR 2.26, 95% CI 1.07–4.77, *p* = 0.009) and SII (OR 2.49, 95% CI 1.22–5.07, *p* = 0.001) are all non-dependent predictors of surgical invasiveness in hip fracture treatment. The first two parameters did not present a convincing sensitivity and specificity. Our finding is in concordance with other research that shows postoperative SII being a more reliable predictor factor than NLR, PLR and MLR [27,28].

Intramedullary gamma nail fixation is a well-recognized minimally invasive technique offering numerous theoretical benefits over plate fixation-based implants, such as lesser biological surgical trauma and greater biomechanical strength [29]. In the USA, during 2000 and 2007, the use of this implant doubled [30]. As noted by Fu et al. [31], approximately 50% of hip fractures are represented by femoral neck fractures, with standard treatment being arthroplasty, as a fixation with other types of implants would represent a malpractice [32]. Many studies compared the clinical outcomes in the elderly population between BHA and unipolar Austin Moore hemiarthroplasty, favoring the first prosthesis due to various reasons such as a lower incidence of thigh pain and better hip function [33]. The duration of surgery (*p* < 0.0001), preoperative days (*p* = 0.022)**,** length of hospital stay (*p* = 0.008) and the ASA score (*p* = 0.028) were relevant results differentiating the two groups of treatment in our study. Previous studies used this kind of measurement in determining the invasiveness of surgical procedures. For example, Salma et al. [34] used the LHS, blood loss and operative time for the orthognathic surgery magnitude measurement. An indirect finding that was also demonstrated by Harper et al. [35] showed a higher blood loss for extracapsular fractures compared to intracapsular fractures, suggested by the RBC levels (*p* < 0.0001) and HGB levels (*p* < 0.0001).

It is well known that immune dysfunction can emerge from surgical interventions, traumatic accidents and endogenous trauma like strokes [36]. Grzelak et al. [37] showed that even a minor procedure like a cholecystectomy can produce a rise in systemic cytokines, including interleukin (IL)-1α, IL-1β, IL-6, IL-8, IL-10 and IFN-γ. In addition, one study [38] compared two procedures performed for anatomic lung resection in patients with malignant cancer, taking into consideration 11 types of cytokines. The researchers concluded that the plasma levels of interleukin (IL)-6 and the monocyte chemo-attractant protein-1 (MCP-1) were higher for the video-assisted thoracic surgery (VATS) group than for the robotic-assisted thoracic surgery (RATS) group. More specific to our domain of musculoskeletal trauma, Reikeras et al. [39] concluded that patients undergoing total hip replacement (THA) presented higher levels of the IL-6, IL-8 and IL-16 cytokines and lower levels in the IL-12 cytokine and Eotaxin.

The routine blood-derived biomarkers (NLR, PLR and SII) have been extensively studied as independent factors in predicting one-year mortality post hip fractures in older patients that benefit from surgical treatment [40,41,42]. A recent systematic review of eight national registries and 36 countries presented a 22% one-year death rate [43].

From this perspective, the findings of our study particularly emphasize the predictive value of the markers we have studied, which are sensitive enough to indicate a correlation with general inflammation in the body. This condition is simultaneously indicated by pro-inflammatory cytokines as well as by the markers indicated by us, and therefore, both categories of laboratory investigations are not absolutely necessary. Also, the markers explored in this research were previously correlated with various pathological conditions such as end-stage kidney disease, systemic lupus erythematosus (SLE), ankylosing spondylitis (AS), rheumatoid arthritis (RA), COVID-19 infection, etc.

Our findings are also important from an economic perspective. Unlike the pro-inflammatory cytokines, which require a separate medical prescription and kits for laboratory testing, and are completed with supplementary charges, the markers studied by us are measured from the current blood analyses performed when the patient is hospitalized and postoperatively as a basic investigation and do not require additional costs. Regarding the duration of the laboratory analysis, the immunoassays require a longer investigation period of at least 24 h or more depending on the type of cytokine, compared to the indexes studied by us, for which the results are obtained in a few minutes.

Hence, the first limitation regarding the design of our monocentric retrospective study can be improved by enhancing the research to a prospective multicentric design. Secondly, CBCs are routinely performed, and there are no standardized protocols to include other inflammatory markers like the C-reactive protein that can be studied. Also, in this research, pro-inflammatory cytokines were not studied, as an enzyme-linked immunoassay (ELISA) is not commonly performed. Finally, blood tests were not repeated at discharge in all cases, and performing blood tests at discharge could provide additional information on the evolution of these biomarkers.

## 5. Conclusions

This study evaluated the relation between the predictiveness of the inflammatory indexes and the trauma of orthopedic surgeries sustained by an age risk category group of patients who are predisposed to hip fractures. The postoperative SII > 1564.7 and the duration of surgery >60.5 min are strongly correlated to the invasiveness of the surgical procedure in the elderly. SII as a systemic biomarker may have a role in clinical practice for the field of orthopedic surgery, but additional studies are needed to establish its value.

## Figures and Tables

**Table 1:** Optimum cut-off values identified using an ROC curve analysis.

Variables	Cut-off Values	AUC	95% CI	*p*-Value
NLR—Admission	8.9	0.47	0.37–0.57	0.551
PLR—Admission	217.5	0.48	0.38–0.58	0.799
MLR—Admission	0.6	0.40	0.30–0.50	0.057
SII—Admission	1606.5	0.47	0.37–0.57	0.627
NLR—Postoperative	8	0.57	0.47–0.67	0.156
PLR—Postoperative	185.3	0.58	0.47–0.68	0.119
MLR—Postoperative	0.8	0.47	0.37–0.57	0.637
SII—Postoperative	1564.7	0.60	0.50–0.70	0.038
Preoperative days	2.5	0.59	0.49–0.69	0.067
Duration of surgery (minutes)	60.5	0.91	0.86–0.96	<0.0001
Length of hospital stay (days)	7.5	0.66	0.56–0.75	0.001

Abbreviations: ROC—receiver operating characteristic; AUC—area under the curve; CI—confidence interval; NLR—neutrophil-to-lymphocyte ratio; PLR—platelet-to-lymphocyte ratio; MLR—monocyte-to-lymphocyte ratio; SII—systemic inflammatory index. *p*-value < 0.05 was considered statistically significant.

**Table 2:** Baseline characteristics, surgery-related data and pre- and postoperative laboratory data. The patients were divided according to the anatomic location of the fracture.

Variable	Total Patients (*n =* 129)	Extracapsular Fracture Group (*n =* 67)	Intracapsular Fracture Group (*n =* 62)	*p*-Value
Baseline characteristics				
Age (years), median (IQR)	81 (9)	81 (8)	80.50 (12)	0.470
Sex, *n* (%)				0.844
Male	35 (27.1)	19 (28.4)	16 (25.8)	
Female	94 (72.9)	48 (71.6)	46 (74.2)	
Alcohol (yes), *n* (%)	34 (26.4)	23 (34.3)	11 (17.7)	0.045
Tobacco (yes), *n* (%)	33 (25.6)	20 (29.9)	13 (21)	0.313
Obesity (yes), *n* (%)	64 (49.6)	32 (47.8)	32 (51.6)	0.726
Living area, *n* (%)				0.157
Rural	59 (45.7)	35 (52.2)	24 (38.7)	
Urban	70 (54.3)	32 (47.8)	38 (61.3)	
HBP (yes), *n* (%)	106 (82.2)	57 (85.1)	49 (79)	0.491
COPD (yes), *n* (%)	37 (28.7)	18 (26.9)	19 (30.6)	0.699
CVI (yes), *n* (%)	40 (31.0)	24 (35.8)	16 (25.8)	0.256
CHF (yes), *n* (%)	71 (55.0)	39 (58.2)	32 (51.6)	0.483
CKD (yes), *n* (%)	21 (16.3)	9 (13.4)	12 (19.4)	0.475
DM (yes), *n* (%)	22 (17.1)	9 (13.4)	13 (21)	0.349
Surgery-related data				
ASA score, *n* (%)				0.028
I–II	34 (26.4)	12 (17.9)	22 (35.5)	
≥III	95 (73.6)	55 (82.1)	40 (64.5)	
Type of anesthesia, *n* (%)				0.818
Intraspinal	106 (82.2)	56 (83.6)	50 (80.6)	
General	23 (17.8)	11 (16.4)	12 (19.4)	
Preoperative days,				0.022
0–2.5 cut-off	72 (55.8)	44 (65.7)	28 (45.7)	
>2.5	57 (44.2)	23 (34.3)	34 (54.8)	
LHS (days),				0.008
0–7.5 cut-off	62 (48.1)	40 (59.7)	22 (35.5)	
>7.5	67 (51.9)	27 (40.3)	40 (64.5)	
Duration of surgery (min),				<0.0001
0–60.5 cut-off	65 (50.4)	57 (85.1)	8 (12.9)	
>60.5	64 (49.6)	10 (14.9)	54 (87.1)	
Admission laboratory data				
Neutrophil count (×10^3^/µL), median (IQR)	8.35 (4.28)	8.35 (3.67)	8.09 (4.91)	0.925
Lymphocyte count (×10^3^/µL), median (IQR)	1.21 (0.66)	1.17 (0.57)	1.21 (0.77)	0.578
Monocyte count (×10^3^/µL), median (IQR)	0.72 (0.36)	0.76 (0.31)	0.69 (0.41)	0.038
PLT count (×10^3^/µL), median (IQR)	220 (88)	225 (114)	217.5 (82)	0.891
AST/ALT (>1, reference), median (IQR)	1.23 (0.56)	1.21 (0.57)	1.26 (0.56)	0.934
WBC (×10^3^/µL), median (IQR)	10.05 (4.52)	10.2 (3.94)	9.47 (5.02)	0.810
RBC (×10^6^/µL), mean ± SD	4.02 ± 0.67	3.76 ± 0.61	4.3 ± 0.62	<0.0001
HGB (g/dL), mean ± SD	12.26 ± 1.84	11.61 ± 1.87	12.97 ± 1.52	<0.0001
NLR (>8.9, cut-off), *n* (%)	44 (43.1)	21 (31.3)	23 (37.1)	0.578
PLR (>217.5, cut-off), *n* (%)	45 (34.9)	21 (31.3)	24 (38.7)	0.460
MLR (>0.6, cut-off), *n* (%)	69 (53.5)	39 (58.2)	30 (48.4)	0.293
SII (>1606.5, cut-off), *n* (%)	58 (45)	29 (43.3)	29 (46.8)	0.726
Preoperative laboratory data				
Neutrophil count (×10^3^/µL), median (IQR)	6.79 (3.69)	3.37 (6.49)	4.38 (7.79)	0.047
Lymphocyte count (×10^3^/µL), median (IQR)	1.13 (0.71)	0.79 (1.21)	0.69 (1.08)	0.546
Monocyte count (×10^3^/µL), median (IQR)	0.82 (0.47)	0.40 (0.85)	0.47 (0.73)	0.051
PLT count (×10^3^/µL) mean ± SD	224.12 ± 70.32	217.21 ± 71.90	231.60 ± 68.32	0.247
WBC (×10^3^/µL), median (IQR)	9.17 (4.31)	8.67 (3.49)	9.53 (4.53)	0.172
RBC (×10^6^/µL), mean ± SD	3.36 ± 0.65	3.11 ± 0.59	3.63 ± 0.61	<0.0001
HGB (g/dL) mean ± SD	10.24 ± 1.85	9.53 ± 1.66	11 ± 1.75	<0.0001
NLR (>8, cut-off), *n* (%)	44 (34.1)	17 (25.4)	27 (43.5)	0.041
PLR (>185.3, cut-off), *n* (%)	64 (49.6)	26 (38.8)	38 (61.3)	0.014
MLR (>0.8, cut-off), *n* (%)	54 (41.9)	27 (40.3)	27 (43.5)	0.725
SII (>1572.7, cut-off), *n* (%)	59 (45.7)	18 (26.8)	41 (66.1)	0.012

Abbreviations: HBP—high blood pressure; COPD—chronic obstructive pulmonary disease; CVI—chronic venous insufficiency; CHF—congestive heart failure; DM—diabetes mellitus; ASA score—American Society of Anesthesiologists score; PLT—platelet; AST—aspartate aminotransferase; ALT—alanine transaminase; WBC—white blood count; RBC—red blood count; HGB—hemoglobin; NLR—neutrophil-to-lymphocyte ratio; PLR—platelet-to-lymphocyte ratio; MLR—monocyte-to-lymphocyte ratio; SII—systemic immune-inflammation index. *p*-value < 0.05 was considered statistically significant.

**Table 3:** Multivariate analysis of surgery-related trauma.

Variable	Surgery-Related Trauma		*p*-Value
	OR	95% CI	
Postoperative NLR	1.79	0.88–3.64	0.029
Postoperative PLR	2.26	1.07–4.77	0.009
Postoperative MLR	1.14	1.22–5.070	0.063
Postoperative SII	2.49	1.22–5.07	0.001
Duration of surgery	38.47	14.13–104.73	<0.0001
Type of anesthesia	1.22	0.49–3.01	0.424
Alcohol	0.41	0.18–0.94	0.199
Tobacco	0.62	0.27–1.39	0.371
Obesity	1.16	0.58–2.32	0.390
HBP	0.66	0.26–1.64	0.123
Asthma and COPD	1.20	0.56–2.58	0.081
CVI	0.62	0.29–1.32	0.886
CHF	0.76	0.38–1.53	0.556
CKD	1.54	0.60–3.97	0.236
DM	1.71	0.67–4.33	0.985

Abbreviations: NLR—neutrophil-to-lymphocyte ratio; PLR—platelet-to-lymphocyte ratio; MLR—monocyte-to-lymphocyte ratio; SII—systemic immune-inflammation index; HBP—high blood pressure; COPD—chronic obstructive pulmonary disease; CVI—chronic venous insufficiency; CHF—congestive heart failure; DM—diabetes mellitus; ASA score—American Society of Anesthesiologists score. *p*-value < 0.05 was considered statistically significant.

## Data Availability

The data used in this study can be requested from the corresponding authors.
